# Decisions About Suppressive Antibiotics Among Clinicians at Veterans Affairs Hospitals After Prosthetic Joint Infection

**DOI:** 10.1001/jamanetworkopen.2025.1152

**Published:** 2025-03-19

**Authors:** Kimberly C. Dukes, Julia Friberg Walhof, Stacey Hockett Sherlock, Dan Suh, Poorani Sekar, Hiroyuki Suzuki, Heather Schacht Reisinger, Bruce Alexander, Kelly Richardson Miell, Brice Beck, Andrew Pugely, Marin L. Schweizer

**Affiliations:** 1 Center for Access & Delivery Research & Evaluations (CADRE), Iowa City Veterans Affairs Health Care System, Iowa City, Iowa; 2 Department of Internal Medicine, University of Iowa Carver College of Medicine, Iowa City; 3 College of Public Health, University of Iowa, Iowa City; 4 Institute for Clinical and Translational Science, University of Iowa, Iowa City; 5 Department of Surgery, University of Iowa Carver College of Medicine, Iowa City; 6 William S. Middleton Veterans Affairs Hospital, Madison, Wisconsin; 7 Department of Internal Medicine, School of Medicine and Public Health, University of Wisconsin, Madison

## Abstract

**Importance:**

Indefinite suppressive antibiotic therapy (SAT) is sometimes prescribed after initial antibiotic treatment for prosthetic joint infection (PJI). Limited evidence on outcomes after SAT exists, and using SAT for patients at low risk who may not need it could be associated with antibiotic resistance and adverse events.

**Objectives:**

To characterize clinical decision-making about SAT after PJI and identify stewardship intervention opportunities to stop or reduce SAT for patients who may not benefit.

**Design, Setting, and Participants:**

In this qualitative study, interviews were conducted with 41 clinicians involved in decision-making about SAT after PJI at 8 US Veterans Affairs hospitals between November 1, 2019, and July 31, 2021. Analysis was conducted from June 9, 2020, to August 31, 2022.

**Main Outcomes and Measures:**

Systematic thematic analysis of transcripts of semistructured interviews was conducted to assess the decision-making process for SAT after PJI, including identifying decision-makers, risks and benefits of SAT, and significant time points that occur before or after the SAT prescribing decision.

**Results:**

A total of 41 clinicians were interviewed. Interviewees reported a complex, usually patient-specific, sometimes collaborative decision-making process. Decisions were emotionally charged because of serious possible repercussions for patients and limited evidence about benefits and risks associated with SAT. Surgeons and infectious diseases physicians were the primary SAT prescribers. Their initial risk-benefit calculation for SAT usually included whether revision surgery could be performed and what type, the organism, patient factors, and clinical signs of infection, as well as their perception of the existing evidence base for SAT after PJI. Interviewees identified significant time points that occured before or after the SAT prescribing decision, including PJI treatment decisions and follow-up appointments. Other potential decision-makers over time included patients, primary care physicians, and pharmacists. Interviewees identified opportunities to discuss SAT-associated benefits and risks with patients as well as other clinicians. Interviewees wanted more evidence about patient outcomes to inform prescribing decisions and emphasized the importance of clinician autonomy and buy-in for practice change.

**Conclusions and Relevance:**

This qualitative study found that surgeons and infectious diseases physicians often made initial decisions about SAT and identified other potential decision-makers (patients, primary care physicians, pharmacists) and significant time points that occur before or after the SAT prescribing decision, including PJI treatment decisions and follow-up appointments. Stewardship interventions should take into account decision points for patients with PJI across time and the range of decision-makers, including patients, across time.

## Key Points

**Question:**

How do clinicians make decisions about suppressive antibiotic therapy for patients who had prosthetic joint infections (PJIs) when there is limited evidence about benefits and risks?

**Findings:**

This qualitative study found that surgeons and infectious diseases physicians often made initial decisions about suppressive antibiotic therapy; other decision-makers over time included patients, primary care physicians, and pharmacists. Clinicians identified significant time points that occur before or after the suppressive antibiotic therapy prescribing decision, including PJI treatment decisions and follow-up appointments.

**Meaning:**

Suppressive antibiotic therapy stewardship interventions should be made with awareness of decision points for patients with PJI across time as well as the range of potential decision-makers, including patients, across time.

## Teaser

This qualitative study characterizes clinical decision-making about suppressive antibiotic therapy after prosthetic joint infection and identifies stewardship intervention opportunities to stop or reduce suppressive antibiotic therapy for patients who may not benefit.

## Introduction

Prosthetic joint infections (PJIs) after hip, knee, or shoulder replacement surgery can have profound health and economic consequences.^1,2,3,4^ Treatment options include surgical revision and antibiotic therapy.^5^ Patients who develop PJI may be prescribed indefinite suppressive antibiotic therapy (SAT; defined here as >6 months of antibiotics) after the initial antibiotic treatment for the PJI, sometimes for life.^6,7^ However, there is limited evidence on outcomes after SAT.^7,8,9^ Using SAT for patients at low risk who may not need it could be associated with antibiotic resistance and adverse events, such as *Clostridioides difficile* infection.^8,10^ Deimplementing SAT—whether that means not prescribing or reducing patients’ length of SAT—for patients at low risk of PJI recurrence may reduce the emergence of antibiotic-resistant organisms as well as the risks associated with long-term antibiotics for those patients.

National agencies currently prioritize reducing the use of unneeded antibiotics and thus reducing antibiotic-resistant bacteria.^11,12^ Guidelines from the Infectious Diseases Society of America do not provide unanimous recommendations about which patients should receive SAT after PJI treatment or the optimal duration for SAT.^13^ Surgeons and other clinicians experiencing stress when patients develop PJIs^14,15,16^ seek the best evidence for PJI treatment.^17^ Thus, understanding the current practice of SAT prescribing for patients with PJI is crucial to developing interventions to optimize SAT for patients who may not benefit from it.

As part of a larger study investigating SAT prescribing and patient outcomes across the Veterans Affairs (VA) health care system, this qualitative study aimed to characterize clinical decision-making processes about SAT prescribing for patients with PJI and identify potential opportunities for antibiotic stewardship interventions to stop SAT prescribing or reduce its duration for patients at low risk.

## Methods

We conducted interviews with clinicians at 8 VA hospitals between November 1, 2019, and July 31, 2021. From 93 VA hospitals that treat patients with PJI, we purposefully sampled hospitals with the goal of maximizing variability in SAT treatment practices (numbers of debridement vs 2-stage procedures after PJI, days of antibiotic treatment) and other hospital characteristics (eg, region, patient volume, hospital complexity, presence of infectious diseases [ID] physicians). Two hospitals declined to participate; for each of those hospitals, we identified another institution with a similar distribution of factors. This study was approved by the University of Iowa institutional review board and the Iowa City VA Research and Development Committee as human participants research. Participants provided oral consent. The study received a waiver of documentation of consent to ensure confidentiality for participants discussing sensitive matters. We report additional details about the methods according to the Consolidated Criteria for Reporting Qualitative Research (COREQ) reporting guideline.^18^

If a hospital agreed to participate, we asked surgical or ID leaders to identify orthopedic surgeons, ID physicians, advanced practice clinicians, nurses, or case managers who were involved with SAT prescribing after PJI and to identify whether there were individuals in other roles who made SAT prescribing decisions or contributed information to help make those decisions. Additional roles identified by individual hospitals (eg, pharmacist, primary care physician [PCP]) were not recruited at other hospitals unless also identified at those hospitals. We invited all identified personnel to participate in in-depth interviews.

We prospectively developed and piloted semistructured, role-focused interview guides with open-ended questions. Interview domains included PJI treatment and SAT prescribing practices and rationales (eg, policies, patient factors, concerns about medical liability, potential antibiotic-related adverse events, information about infection); patient and clinician involvement, including shared decision-making; perceptions about potential interventions to optimize prescribing; and organizational factors. (We provide an example interview guide in the eAppendix in Supplement 1.) Trained qualitative researchers (K.C.D. and J.F.W.) conducted interviews in person during site visits, by telephone, or over virtual platforms. We digitally audio-recorded interviews with oral interviewee consent. Recordings were then transcribed verbatim and imported into MAXQDA software, version 18,20,22,^19^ to facilitate coding and analysis.

A multidisciplinary team (K.C.D., J.F.W., S.H.S., D.S., P.S., H.S., and M.L.S.) conducted consensus-based thematic analysis of transcripts.^20,21,22^ Using collaborative discussion of 8 individually coded transcripts, the team developed a thematic codebook using both deductive and inductive analysis. We iteratively adapted the codebook during preliminary analyses, systematically documented codebook changes and their rationales, and ensured that final codes were applied to all transcripts. Subsequently, 2 team members (K.C.D. and J.F.W.) coded transcripts individually and conducted comparisons within roles and within sites (eg, comparing themes by surgeon roles or within hospitals). We held periodic team reviews to ensure codebook fidelity, resolve any ambiguities, and assess whether we had achieved data saturation. Analysis was conducted from June 9, 2020, to August 31, 2022.

## Results

We interviewed 41 clinicians at 8 VA hospitals with roles in prescribing SAT to patients who had experienced PJI (Table 1). Participating hospitals operated in 7 states and represented 6 of the VA’s 18 regional Veterans Integrated Service Networks, across the US Southeast, Southwest, Middle Atlantic, and Midwest. The complexity level of these hospitals is included in Table 2. Two hospitals declined to participate. The number of respondents from each hospital varied from 2 to 11. We provide exemplar quotations in the Box and reference them by number in the Results.

**Table 1.** Interviewee Roles

Role	No. of interviewees (N = 41)
Infectious diseases physician	14
Orthopedic surgeon	12
Advanced practice clinician	5
Nurse	6
Primary care physician	1
Pharmacist	1
Infection preventionist	1
Hospitalist	1

**Table 2.** Participating Hospitals by Complexity Level

Hospital	Complexity level^a^
1	1c
2	1c
3	1c
4	1a
5	1a
6	1a
7	1a
8	1a

^a^ Complexity level: 1a indicates highest complexity; and 1c, mid-high complexity.

Box. **Exemplar Quotations Associated With Presented Themes^a^**

**Decision-Making About PJI Treatment and Subsequent Initial SAT Prescribing**

- 1. … [I recommend SAT for patients] who have had multiply revised, multiply failed hip or knee replacements with multiple infections. And also those patients have to be poor hosts, and by poor hosts, I mean medical comorbidities that make it so that they probably won’t ever clear their infection… (Surgeon, hospital 6)
- 2. Surgeons that take care of PJIs, …and see the devastation of that problem are naturally going to be bent toward “I’m going to do everything in the world to prevent this and I don’t care that we’re changing the antibiotic profile around the world by using too many antibiotics.” (Surgeon, hospital 3)
- 3. [For example, after a MRSA culture result] we’ll have a conversation and say, “Hey, I don’t feel we can eradicate this infection if you just take out, you know, some of the hardware, not all of it.” (ID physician, hospital 4)

**Decision-Making Among Clinicians About SAT After Initial PJI Treatment: Surgeons, ID Physicians**

- 4. I would take what ID recommends. (Surgeon, hospital 1)
- 5. Let the surgeon kind of stay in their realm—they cut. (ID physician, hospital 1)
- 6. Sometimes we have some really gray cases so if it’s not clear on the guidelines, usually we discuss it as a group among the ID team, on how we would approach the situation and also work with their, like their orthopedic surgeons too. (Pharmacist, hospital 6)
- 7. It’s their patient so they [surgeons] can decide to do whatever they want. I can’t stop them from giving it [antibiotics] if that’s what they think is appropriate. (ID physician, hospital 3)
- 8. As a surgeon, my job is to do surgery; and just managing medications, there’s other physicians for that. (Surgeon, hospital 6)
- 9. Usually we will do primarily what the ID folks say because you know, that’s their job, they’re the experts there. (Nurse practitioner, hospital 4)

**Decision-Making on SAT Continuation: Primary Care, Patients, Pharmacists, ID Physicians, Surgeons**

- 10. [Eventually, PCPs] end up making the decision about, a lot of times, like is this [SAT] really going to continue forever or not. (PCP, hospital 1)
- 11. [Most patients who stop] do it by accident; they forget why they’re taking them or someone stops the antibiotic, saying “You shouldn’t be on chronic antibiotics, do you know why you are?” They don’t know or they have forgotten. (ID physician, hospital 4)
- 12. Some patients kind of self-select; they just say, “You know I’m tired of these antibiotics. I want to trial without it.” (ID physician, hospital 8)
- 13. PCPs actually are very nervous about dealing with antibiotics and so if there’s some issue, if there’s any kind of concern then they will reach out to us about what to do. (ID physician, hospital 6)
- 14. [Occasionally] I have pushed it [SAT] on the patient, and they have tried it, but then without my knowledge aborted it because of severe gastrointestinal side effects, rashes, whatever, you know? (Surgeon, hospital 4)

**Shared Decision-Making With Patients**

- 15. [Patients] have to have skin in the game. They’re the ones who are going to be taking antibiotics every day for the rest of their life. (Surgeon, hospital 6)
- 16. Well, usually the patient will do whatever I want to do, so if there’s a disagreement, probably just do what I recommend them. (Surgeon A, hospital 5)
- 17. By and large, I’d say the 4 main players are the surgeon, the patient, ID, and primary care, with ultimate decision—the final decision being made jointly between the patient and the surgeon. (Surgeon, hospital 4)
- 18. …[T]o make an informed decision with the patient you need to know like, “Okay well, if we stop this there’s a 20% chance it’ll come back or a 5% chance.” It’s really hard for me to give that information to the patient ‘cause we don’t really know. So it’s typically speaking generalities, which if I was the patient would be pretty frustrating to me. (ID physician, hospital 3)
- 19. [After 6 months, regularly assessing the patient’s tolerance to SAT] I do explain to them [patients] that you know, as we get further out, there’s less benefit and that ratio changes. (ID physician, Hospital 7)

Abbreviations: ID, infectious diseases; MRSA, methicillin-resistant *Staphylococcus aureus*; PCP, primary care physician; PJI, prosthetic joint infection; and SAT, suppressive antibiotic therapy.

^a^
Lightly edited for clarity.

### Summary of Initial Decision-Making Process

Interviewees reported a complex, usually patient-specific, sometimes collaborative decision-making process. The process varied by hospital and within hospitals and sometimes by clinician. Through interview data, we identified a range of potential antibiotic decision-makers: orthopedic surgeons, ID physicians, PCPs, pharmacists, and patients. The involvement of these decision-makers over time will be described in the following section.

### An Extended Timeline of Decision Points Relevant to Prescribing or Maintaining SAT After PJI

We found that SAT decision-making could be a long process with multiple opportunities for decisions, rather than a 1-time decision, as we initially assumed. Rather than a single time point at which SAT is prescribed or not prescribed, interview data identified multiple points at which decisions are made that will shape the possibilities of SAT being prescribed, continued, or stopped. Interviewees also identified other significant time points that occurred before or after the SAT prescribing decision (Figure). Thus, interview data gathered across roles allowed us to recognize an extended timeline that includes a formal decision to prescribe SAT but extends backward and forward from that moment. In our analysis, the extended timeline includes the following time points: A, initial decision about PJI treatment; B, the SAT prescribing decision, which occurred after initial intravenous and oral antibiotic treatment for the PJI; and C, D, E, and beyond (follow-up appointments where SAT could be continued or discontinued, intentionally or unintentionally). Multiple people may participate in the decision at each point.

**Figure.**
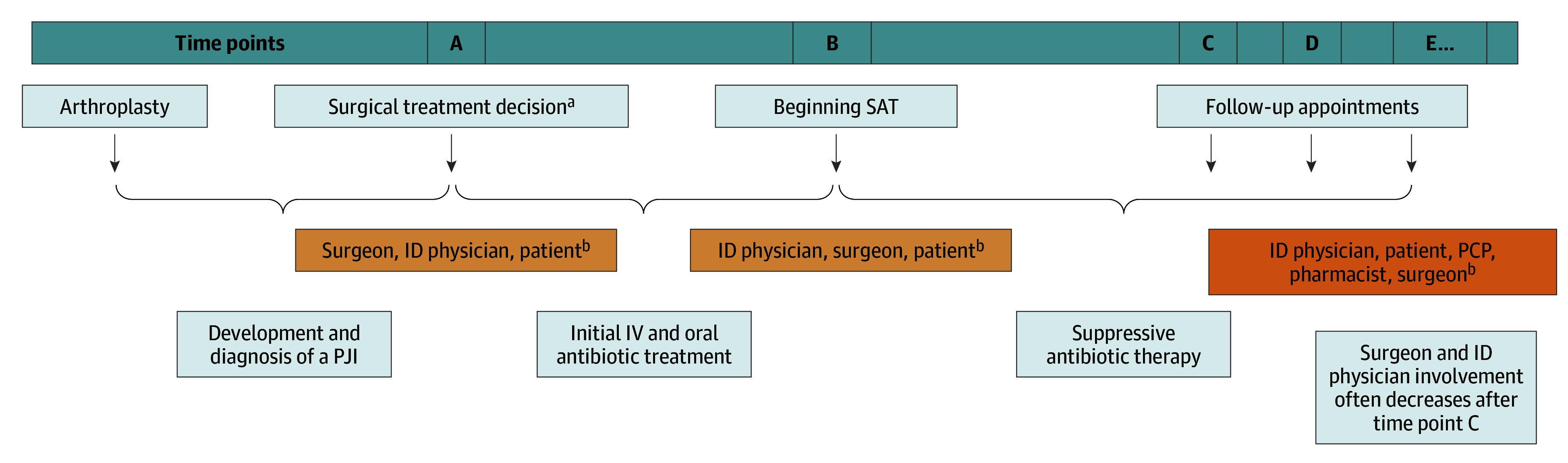
Timeline of Decision Points Relevant to Prescription or Maintenance of Suppressive Antibiotic Therapy (SAT) After Prosthetic Joint Infection (PJI) ID indicates infectious diseases; IV, intravenous; and PCP, primary care physician. ^a^Such as debridement, 1- or 2-stage exchange, or no surgery. ^b^Degree of collaboration varies and may include others.

#### Time Point A

Our first identified time point is the initial decision by the surgeon about PJI treatment, including whether or not the patient was deemed eligible for revision surgery, months before a SAT prescribing decision could be made. Interviewees often pointed out that these treatment decisions shaped and circumscribed potential future SAT prescribing decisions.

At this point, several surgical treatment options, including debridement and retention and 1- or 2-stage exchange, are available. At this time point, surgeons sometimes involved ID physicians, but not always. At time point A and beyond, interviewees often described that surgeons and ID physicians both aimed for best outcomes for patients, but they tended to focus on different risks for the patient. For example, ID physicians sometimes reported that they hoped to encourage surgeons to be more aggressive in surgical revision (eg, quotation 3 in the Box) and might worry about adverse events and antibiotic resistance, while surgeons felt they had unique knowledge about the type of revision they thought was possible for the patient and worried more about further PJIs than antibiotic-related adverse events (eg, quotations 1 and 2 in the Box). ID physicians and others reported valuing surgeons’ knowledge of the surgical possibilities for each patient.

At time point A, patients were sometimes involved in this discussion (eg, quotations 16 and 17 in the Box), and interviewees reported that sometimes patients made choices that might differ from the clinical recommendation. Two interviewees reported that patients might reject amputation as a surgical option, for example, which could influence whether or not the interviewee would prescribe SAT at time point B.

#### Time Point B

The second time point on the timeline is the SAT prescribing decision, which occurred after initial intravenous and oral antibiotic treatment for the PJI.

##### Involvement in and Processes for Initial SAT Decisions

Across hospitals, interviewees reported that surgeons and ID physicians were the primary initial SAT prescribers. At some hospitals, surgeons took the lead; at others, surgeons deferred to ID physicians, or surgeons and ID physicians collaborated closely (eg, quotations 4-9 in the Box). In addition, sometimes these 2 sets of prescribers consulted pharmacists or antibiotic stewardship team members, either about overall prescribing policy or prescribing for individual patients (eg, quotation 6 in the Box). Across hospitals, interviewees reported that surgeons or ID physicians sometimes conducted shared decision-making with patients. Some interviewees pointed out that advanced practice clinicians and nurses also contributed valuable patient information to inform decisions.

Interviewees at 3 hospitals reported that surgeons relied on ID physicians’ expertise in SAT decision-making, although one of these hospitals did not have ID physicians available every day. At a different hospital, surgeons made all SAT decisions and only consulted ID physicians if there were ambiguity or questions about the infecting organism. Interviewees at 1 hospital reported variability regarding whether the surgeon or the ID physician made SAT decisions. At another hospital, 1 surgeon reported consulting internal medicine physicians in addition to ID physicians regarding SAT. Interviewees at other hospitals reported collaboration and consultation, but surgeons made final decisions.

At all hospitals, interviewees reported some degree of collaboration between orthopedic surgeons and ID physicians. However, the timing and degree of collaboration varied among hospitals, as did the availability of ID physicians and the influence of each specialty. A few interviewees reported collaborations with other partners (eg, pharmacists, antibiotic stewards) or occasionally PCPs. Shared decision-making with patients was valued (eg, quotation 15 in the Box) but variably implemented, and interviewees described barriers to shared decision-making about SAT, including the extended time it would take to discuss the complexity of the risk-benefit assessment with a patient.

##### Policies and Considerations

When asked about policies guiding SAT prescribing after PJI, very few clinicians reported having a policy. Rather, they tended to make patient-specific decisions based on their perceptions of the risk of PJI recurrence. Some interviewees prescribed SAT for all patients after PJI revision surgery. Interviewees reported that post-PJI SAT decisions are often difficult, and they often expressed deep concern about possible serious repercussions for patients who might experience recurrent PJI. They also often felt that the evidence available to them about outcomes associated with SAT was limited, ambiguous, or did not apply adequately to their concerns about individual patients. Thus, for those who did not have a consistent policy, their decision-making about SAT could be emotionally charged.

In discussing rationales for prescribing SAT, surgeons often described the worry that if SAT was not prescribed, their patients would have worse outcomes if they experienced recurring PJI or reported that they considered whether the patient would be a candidate for further surgery. Surgeons told stories of patients’ severe pain or other adverse experiences after PJI and wanted to avoid this outcome for their patients. They seldom described considering potential drawbacks to long-term antibiotic regimens. One surgeon, for example, pointed out that due to their specific role, they would seldom personally see any patient who was experiencing repercussions from long-term antibiotics but would encounter the adverse experiences of the patient with a recurring PJI.

Interviewees reported that their initial risk-benefit calculation for SAT usually included whether revision surgery was performed and what type, the infecting organism, patient preference and patient factors, and clinical signs of infection. They incorporated their perception of the evidence base, which many found insufficient. Interviewees described weighing both clinical and social factors in their prescribing decisions for individual patients. Reported clinical factors included age, comorbidities, immunosuppression, prior revisions or replacements, the amount of bone left for future surgical procedures, and the patient’s tolerance of antibiotic treatment. Reported social factors included the patient’s comprehension of the risks and benefits associated with SAT, as well as the patient’s social situation, residence, and projected compliance. Finally, the interviewees considered how to best maintain patient mobility and quality of life. This concern included considerations regarding amputation. Interviewees assessed the likelihood of amputation as well as patients’ amputation survival risk. Several interviewees described that if they thought eventual amputation might be needed, they hoped to delay amputation when possible and thought SAT might help extend a delay.

When asked about previously identified barriers to appropriate prescribing of SAT (eg, insufficient time with each patient, diagnostic uncertainty, and liability concerns), interviewees generally did not report worrying about these. Interviewees did report being risk averse in their decision-making.

#### Time Points C, D, E, and Beyond

Once the initial SAT prescribing decision had been made, time points C, D, E, and beyond occurred in the extended follow-up period. This follow-up period itself had multiple time points and could extend over years. Communication and prescribing responsibility varied throughout the extended follow-up time points. Interviewees reported that surgeons and ID physicians were often heavily involved in time points A, B, and C and that their involvement lessened in later phases of the follow-up. At time point C, surgeons and ID physicians often conducted initial follow-up visits with patients receiving SAT regimens, with ID physicians reporting longer periods of follow-up. However, at some point, surgeons and ID physicians stopped following up with a patient; this time point varied among the 8 hospitals. We call this time point D.

During time points C, D, E, and beyond of a patient’s SAT regimen, other decision-makers played significant roles at different times. These decision-makers included patients, PCPs, and pharmacists, especially after surgeons or ID physicians stopped following up with patients. When the patient had progressed to long-term follow-up, interviewees reported that PCPs sometimes participated in deciding whether to continue SAT prescribing (eg, quotations 10 and 13 in the Box). At 1 hospital, ID pharmacists had been heavily involved in following up with patients who had been prescribed SAT, with 6-month check-ins.

During the long period of follow-up, starting at time point C, interviewees reported many ways in which patients’ SAT prescriptions might be stopped (eg, quotations 11, 12, and 14 in the Box). The prescription could be stopped intentionally, when a clinician stopped SAT because of adverse effects or because they thought SAT was not helping a patient. Multiple interviewees described adverse effects that led to recommendations to stop SAT for patients. Interviewees suggested that stopping SAT also sometimes occurred without specific clinical intention. Patients sometimes moved or changed clinical practitioners, and the prescription or the rationale for the prescription did not always follow the patient. Patients sometimes decided they did not want to take SAT any longer and stopped on their own. Several interviewees reported that they felt sometimes PCPs were uncomfortable renewing prescriptions or conversely were uncomfortable about stopping the prescription. Interviewees reported that PCPs sometimes had limited opportunities to confer with the original prescriber about the rationale or to review the continuing need for the prescription. Two interviewees also reported that patients’ social situations or modifiable risk factors, such as smoking, could reduce their willingness to recommend stopping SAT.

## Discussion

Little is currently known about how physicians make decisions about prescribing SAT to patients who experience PJI. We began our project focusing on what we thought would be a single decision point to prescribe SAT after initial antibiotic treatment for the PJI. Instead, we learned from interviewees that, while there was 1 point at which the primary SAT prescribing decision was made, many other time points were associated with SAT prescribing decisions. In addition, individuals in different roles were involved as decision-makers at different time points. We identified a number of potential antibiotic decision-makers—orthopedic surgeons, ID physicians, PCPs, pharmacists, patients—whose participation may vary over time, as well as a long timeline for clinical decision-making that could offer opportunities to deimplement SAT prescribing. In addition, we identified significant opportunities for shared decision-making to discuss benefits and risks with patients.

Current orthopedic surgical guidelines provide some guidance about integrating PJI risk factors into SAT management decisions.^23^ These complex decisions are also sometimes emotionally charged because of serious possible repercussions for patients and limited or ambiguous evidence about the benefits and risks associated with SAT. Studies show that orthopedic surgeons have emotional reactions when their patients experience PJI, and their perception of uncertainty may influence the decisions that they make about surgical revisions.^14,15,16,17^ In our study, some surgeons reported that the memories of personal stories of patients who had developed PJI influenced their decision-making because they hoped to protect other patients from similar bad outcomes. However, interviewees also reported that not all patient outcomes were seen by all clinicians. For example, a *C difficile* infection may be treated by a PCP and an ID physician but not a surgeon. There may be an opportunity for antibiotic stewards to inform all clinicians of antibiotic-associated adverse events to assist in comprehensive evaluation of the risks and benefits associated with SAT. In addition, in our study, clinicians felt that they needed better evidence about patient outcomes with or without SAT. To help manage their uncertainty and increase their willingness to forgo prescribing SAT, clinicians could benefit from further scientifically rigorous evidence about outcomes.

In a recent review of the literature about SAT after debridement and implant retention, Cortes-Penfield et al^24^ emphasize that clinicians should thoroughly evaluate risk vs benefits when starting or continuing SAT, including the risks associated with extended antibiotic use, and conduct regular monitoring and review. Recently, Shah et al^8^ suggested that SAT was not associated with benefits after 1 year among patients with knee PJI. In our study, we found that sometimes patients or clinicians stopped SAT without conferring with the original prescribers. We also found that SAT use can end in unexpected ways. Patients move or change clinicians, and new clinicians may not continue the prescriptions, or patients may decide to stop SAT themselves. The original prescriber—whether ID physician or orthopedic surgeon—may have a timeline for SAT duration in mind, but their involvement with the patient’s follow-up decreases over time, and they may not be involved in specific decisions about stopping SAT. The US Centers for Disease Control and Prevention advocates antibiotic time-outs as a point for review of antibiotic therapy in inpatient settings.^25^ Similarly, there may be an opportunity to add specific time points for reflection regarding SAT and identify patients who could stop. Especially because SAT incorporates a very long time period (that may extend to the patient’s lifetime), it might be helpful during initial conversations about prescribing to specify potential end dates or markers for reconsidering the SAT prescription. Antibiotic stewards could identify times for follow-up to facilitate discussions about stopping SAT. Having these set times for review could also assist PCPs in the assessment of the risks or benefits associated with continuing SAT.

Recent reviews of clinical evidence recommend involving patients in shared decision-making about these complex decisions.^24,26^ Interviewees in our study reported variable involvement of patients in shared decision-making about initial surgical treatment of their PJI and subsequent long-term antibiotic suppression. Although many interviewees reported patient involvement as important, some also described barriers to explaining these complex considerations to patients. Stewardship teams may be able to support clinicians in developing clear messaging about known risks and benefits of SAT, as well as what is not currently known.

### Limitations

This study has some limitations. All interviewees worked at VA hospitals in the US, and their concerns about VA patients may not be representative for patients at other types of hospitals. We interviewed clinicians at 8 hospitals but were not always able to talk with all decision-makers at each hospital. We also interviewed only 1 individual in several roles (eg, pharmacist). Because we asked hospitals to identify individuals or roles involved in SAT prescribing decisions and then attempted to recruit them, we did not interview individuals in each role at all hospitals. It is possible that we may have missed identifying elements of the practice of all clinicians at each hospital. Future work could further investigate the potential role of interdisciplinarity in SAT prescribing. In addition, the number of interviewees who agreed to participate at each site varied, due to the topic’s perceived sensitivity as well as pandemic-related barriers. Nevertheless, what we have learned about the overall patterns of SAT prescribing was consistent across participating hospitals and likely is transferable to thinking about what interventions may be acceptable to optimize SAT prescribing in a wider range of hospitals. We did not interview patients. Future work could incorporate patient perspectives on SAT, risk, and shared decision-making.

## Conclusions

This qualitative study found that surgeons and ID physicians often made initial decisions about SAT. We also identified other potential decision-makers (patients, PCPs, pharmacists) and significant time points that occur before or after the SAT prescribing decision, including PJI treatment decisions and follow-up appointments. Patient participation in shared decision-making could be better integrated into all phases of decision-making. In addition, stewardship interventions should recognize the timeline of decision points for patients wth PJI across time and the range of decision-makers across time.

## Notes

Supplement 1. **eAppendix.** Example Interview Guide for VA Physicians and Advanced Practice Providers

Supplement 2.
Data Sharing Statement

## References

[1] Kurtz SM, Lau E, Schmier J, Ong KL, Zhao K, Parvizi J. Infection burden for hip and knee arthroplasty in the United States. J Arthroplasty. 2008;23(7):984-991. doi:10.1016/j.arth.2007.10.017 18534466

[2] Mallon CM, Gooberman-Hill R, Moore AJ. Infection after knee replacement: a qualitative study of impact of periprosthetic knee infection. BMC Musculoskelet Disord. 2018;19(1):352-352. doi:10.1186/s12891-018-2264-7 30285692 PMC6167863

[3] Moore AJ, Blom AW, Whitehouse MR, Gooberman-Hill R. Deep prosthetic joint infection: a qualitative study of the impact on patients and their experiences of revision surgery. BMJ Open. 2015;5(12):e009495. doi:10.1136/bmjopen-2015-009495 26644124 PMC4679895

[4] Andersson AE, Bergh I, Karlsson J, Nilsson K. Patients’ experiences of acquiring a deep surgical site infection: an interview study. Am J Infect Control. 2010;38(9):711-717. doi:10.1016/j.ajic.2010.03.017 21034980

[5] Beam E, Osmon D. Prosthetic joint infection update. Infect Dis Clin North Am. 2018;32(4):843-859. doi:10.1016/j.idc.2018.06.005 30241717

[6] Prendki V, Zeller V, Passeron D, . Outcome of patients over 80 years of age on prolonged suppressive antibiotic therapy for at least 6 months for prosthetic joint infection. Int J Infect Dis. 2014;29(C):184-189. doi:10.1016/j.ijid.2014.09.012 25447723

[7] Siqueira MB, Saleh A, Klika AK, . Chronic suppression of periprosthetic joint infections with oral antibiotics increases infection-free survivorship. J Bone Joint Surg Am. 2015;97(15):1220-1232. doi:10.2106/JBJS.N.00999 26246256

[8] Shah NB, Hersh BL, Kreger A, . Benefits and adverse events associated with extended antibiotic use in total knee arthroplasty periprosthetic joint Infection. Clin Infect Dis. 2020;70(4):559-565. doi:10.1093/cid/ciz261 30944931 PMC7768747

[9] Burr RG, Eikani CK, Adams WH, Hopkinson WJ, Brown NM. Predictors of success with chronic antibiotic suppression for prosthetic joint infections. J Arthroplasty. 2022;37(8S):S983-S988. doi:10.1016/j.arth.2022.02.003 35143924

[10] Malahias MA, Gu A, Harris EC, . The role of long-term antibiotic suppression in the management of peri-prosthetic joint infections treated with debridement, antibiotics, and implant retention: a systematic review. J Arthroplasty. 2020;35(4):1154-1160. doi:10.1016/j.arth.2019.11.026 31955984

[11] Antimicrobial stewardship programs: VHA directive 1031. Department of Veterans Affairs. 2019. Accessed February 8, 2025. https://www.va.gov/vhapublications/ViewPublication.asp?pub_ID=11458

[12] National Academies of Sciences, Engineering, Medicine, Health Medicine Division, Board on Population Health Public Health Practice, Committee on the Long-Term Health Economic Effects of Antimicrobial Resistance in the United States. The National Action Plan for Combating Antibiotic-Resistant Bacteria. In: Palmer GH, Buckley GJ, eds. Combating Antimicrobial Resistance and Protecting the Miracle of Modern Medicine. National Academies Press; 2022:301-320.

[13] Osmon DR, Berbari EF, Berendt AR, ; Infectious Diseases Society of America. Executive summary: diagnosis and management of prosthetic joint infection: clinical practice guidelines by the Infectious Diseases Society of America. Clin Infect Dis. 2013;56(1):1-10. doi:10.1093/cid/cis966 23230301

[14] Mallon C, Gooberman-Hill R, Blom A, Whitehouse M, Moore A. Surgeons are deeply affected when patients are diagnosed with prosthetic joint infection. PLoS One. 2018;13(11):e0207260. doi:10.1371/journal.pone.0207260 30485337 PMC6261566

[15] Walter N, Wimalan B, Baertl S, . Managing periprosthetic joint infection—a qualitative analysis of nursing staffs’ experiences. BMC Nurs. 2022;21(1):190. doi:10.1186/s12912-022-00978-z 35850726 PMC9294832

[16] Svensson K, Rolfson O, Mohaddes M, Malchau H, Erichsen Andersson A. Reflecting on and managing the emotional impact of prosthetic joint infections on orthopaedic surgeons—a qualitative study. Bone Joint J. 2020;102-B(6):736-743. doi:10.1302/0301-620X.102B6.BJJ-2019-1383.R1 32475242

[17] Moore AJ, Blom AW, Whitehouse MR, Gooberman-Hill R. Managing uncertainty—a qualitative study of surgeons’ decision-making for one-stage and two-stage revision surgery for prosthetic hip joint infection. BMC Musculoskelet Disord. 2017;18(1):154-154. doi:10.1186/s12891-017-1499-z 28403859 PMC5388991

[18] Tong A, Sainsbury P, Craig J. Consolidated Criteria for Reporting Qualitative Research (COREQ): a 32-item checklist for interviews and focus groups. Int J Qual Health Care. 2007;19(6):349-357. doi:10.1093/intqhc/mzm042 17872937

[19] MAXQDA 2022 [computer software]. VERBI Software; 2021. Accessed February 8, 2025. https://www.maxqda.com/

[20] Kuckartz U. Qualitative text analysis: a systematic approach. In: Kaiser G, Presmeg N, eds. Compendium for Early Career Researchers in Mathematics Education. Springer International Publishing; 2019:181-197. doi:10.1007/978-3-030-15636-7_8

[21] Garrison DR, Cleveland-Innes M, Koole M, Kappelman J. Revisiting methodological issues in transcript analysis: negotiated coding and reliability. Internet High Educ. 2006;9(1):1-8. doi:10.1016/j.iheduc.2005.11.001

[22] Hemmler VL, Kenney AW, Langley SD, Callahan CM, Gubbins EJ, Holder S. Beyond a coefficient: an interactive process for achieving inter-rater consistency in qualitative coding. Qualitative Res. 2022;22(2):194-219. doi:10.1177/1468794120976072

[23] Diagnosis and prevention of periprosthetic joint infections: evidence-based clinical practice guideline. American Academy of Orthopaedic Surgeons. Accessed February 8, 2025. https://www.aaos.org/globalassets/quality-and-practice-resources/pji/pji-clinical-practice-guideline-final-9-18-19-.pdf

[24] Cortes-Penfield N, Krsak M, Damioli L, . How we approach suppressive antibiotic therapy following debridement, antibiotics, and implant retention for prosthetic joint infection. Clin Infect Dis. 2024;78(1):188-198. doi:10.1093/cid/ciad484 37590953

[25] Core elements of hospital antibiotic stewardship programs. Centers for Disease Control and Prevention. Accessed February 8, 2025. https://www.cdc.gov/antibiotic-use/hcp/core-elements/hospital.html?CDC_AAref_Val=https://www.cdc.gov/antibiotic-use/core-elements/hospital.html 10.1093/cid/ciu542 PMC6521960 25261548

[26] Nelson SB, Pinkney JA, Chen AF, Tande AJ. Periprosthetic joint infection: current clinical challenges. Clin Infect Dis. 2023;77(7):e34-e45. doi:10.1093/cid/ciad360 37434369 PMC11004930

