# Supplementary material for: Decisions About Suppressive Antibiotics Among Clinicians at Veterans Affairs Hospitals After Prosthetic Joint Infection
Source: JAMA Netw Open. 2025 Mar 19;8(3):e251152. doi: 10.1001/jamanetworkopen.2025.1152 (PMC11923720; doi:10.1001/jamanetworkopen.2025.1152)
Supplement: Supplement 1. — eAppendix. Example Interview Guide for VA Physicians and Advanced Practice Providers [file jamanetwopen-e251152-s001.pdf]

Supplementary Online Content

Dukes KC, Walhof JF, Hockett Sherlock S, et al. Decisions about suppressive antibiotics among clinicians at Veterans Affairs hospitals after prosthetic joint infection. *JAMA Netw Open.* 2025;8(3):e251152. doi:10.1001/jamanetworkopen.2025.1152

eAppendix. Example Interview Guide for VA Physicians and Advanced Practice Providers

This supplementary material has been provided by the authors to give readers additional information about their work.

© 2025 Dukes KC et al. *JAMA Network Open.*

eAppendix.

<u>Example Interview Guide for VA Physicians and Advanced Practice Providers</u>

First, would you explain your role in treating patients with prosthetic joint infections?

If you could, walk me through the process of making decisions about sustained antibiotic therapy, or SAT, for patients with prosthetic joint infections in your practice.

Would you tell me more about what comes into play for you as you're making the decision about SAT for an individual patient?

PROBING QUESTIONS (if not introduced by interviewee):

- Are there policies regarding SAT after PJI at your hospital?
- Do concerns about medical liability play into your decision?
- Do potential antibiotic-related adverse events play into your decision?
- Do you feel you always know enough about the infection to make the right decision?
- Do you feel that you have enough literature or evidence to help inform your decision? [Probe further for what the clinician wants.] [Optional depending on time] What would you consider enough?

Given your experience with treating prosthetic joint infections, are there particular patient cases who stump you? What would you want to know about outcomes for those patients? [Optional depending on time] Are there other things you want to know about those patients that could affect your prescribing decisions?

In your practice, is there a role for other providers in making decisions about SAT? (If yes) Could you tell me how that happens?

PROBING QUESTIONS:

- What happens if there is a disagreement?
- Do primary care providers have any role? (if PCP is not introduced by interviewee)

In your practice, do you involve patients in the decision to use SAT? Why or why not?

PROBING QUESTIONS:

- (If yes) Could you tell me how that happens in your practice?
- Do you have enough time with patients to facilitate discussions with them about SAT??

<u>[optional depending on time] Hypothetical Vignettes</u>

Next I'd like to ask about your perspective on a couple of hypothetical vignettes. (Additional vignettes may be developed drawing on examples found during initial chart review.)

In the first vignette, a provider and 55-year old patient engage in shared decision making about the patient's prosthetic joint infection. Shared decision making is a process where clinicians and patients collaborate to make decisions that include consideration of risks, benefits, and patient preferences and values. The clinician feels that the patient is relatively young and healthy, and would be at low risk for a recurrent infection after 2-stage exchange surgery. However, the patient is afraid of the potential consequences of another infection and wants any treatment that would prevent the infection from coming back. **In this case, how would you go about deciding whether to prescribe SAT?**

In the second vignette, the patient had an *S. aureus* PJI and subsequently had a debridement with polyethylene exchange and an antibiotic directed to the staph bacteria, plus rifampin for 6 months. One member of the decision making team recommends against SAT. Another worries that another infection will be catastrophic for the patient. **How do you view this decision making process?**

<u>Potential Interventions</u>

© 2025 Dukes KC et al. *JAMA Network Open.*

Since not all patients benefit equally from SAT, we want to explore potential interventions to optimize SAT prescribing, that is, to limit SAT prescribing to patients who will benefit. [For these questions, probe for more detail based on the description of practice process provided above.]

Are there interventions you think could help to reduce SAT prescribing for patients who might not benefit? If so, what interventions might be helpful?

PROBING QUESTIONS:

- (for each intervention) Would there be barriers to that intervention in your practice?
- (for each intervention) What could make it work?

Ask the following questions if these interventions have not been suggested by interviewee.

Would substituting "watchful waiting" for immediate prescription of SAT work in your practice? Why or why not?

PROBING QUESTIONS:

- Is there anything that would help it work?
- Are there certain groups of patients that you would be willing to try this for?

As we discussed earlier, in shared decision making [share definition above if did not discuss vignettes], clinicians and patients collaborate to make decisions that include consideration of risks, benefits, and patient preferences and values. Would involving a patient in shared decision making work in your practice? Why or why not?

PROBING QUESTIONS:

- Is there anything that would make it work better or worse in your practice?
- Would it be appropriate for a nurse or case manager to talk to the patient about SAT, using shared decision making?
- [If yes] How would you use the feedback from this shared decision making in prescribing decisions?

If an audit and feedback process were in place to provide feedback on concordance with guidelines, how might this work in your practice setting?

PROBING QUESTION:

- What do you see as potential barriers or concerns?
- Can you think of anything that could help it work better?

If an interactive workshop was offered for clinicians to learn the current evidence about which patients may not benefit from SAT prescribing, would this be useful in your practice setting? Why or why not?

PROBING QUESTION:

- From your perspective, what staff should be made aware of such a workshop?
- Are there questions you get from patients that you'd like more information about?
- How do you think an evidence-based workshop like this could best be implemented? (In person, video, etc.)

<u>Organizational Factors</u>

I'd like to ask a few broader questions about the institutional context in which you make decisions about SAT.

Do you think there is a role for hospital leadership in guiding these or similar prescribing decisions?

Are there ways your institution can help support you to reduce SAT use in patients who may not benefit but continue SAT in those who may benefit? (e.g., pharmacy support to counsel patients on SAT's risks)

Has your practice tried any quality improvement initiatives about prescribing practices, including antimicrobial stewardship? Would you tell me about that process?

© 2025 Dukes KC et al. *JAMA Network Open.*

[optional depending on time] Do you have any experience with SAT in the private sector? [If yes] In your experience, is the culture of care around SAT different in the VA?

<u>Concluding</u>

Is there anything else we should be asking about SAT prescribing?

Would you mind if I contacted you in the future if we have additional questions?

Thank you so much for sharing your experience and perspective today.

© 2025 Dukes KC et al. *JAMA Network Open.*
