# Supplementary material for: Decisions About Suppressive Antibiotics Among Clinicians at Veterans Affairs Hospitals After Prosthetic Joint Infection
Source: JAMA Netw Open. 2025 Mar 19;8(3):e251152. doi: 10.1001/jamanetworkopen.2025.1152 (PMC11923720; doi:10.1001/jamanetworkopen.2025.1152)
Supplement: Supplement 2. — Data Sharing Statement [file jamanetwopen-e251152-s002.pdf]

Data Sharing Statement

Dukes. Decisions About Suppressive Antibiotics Among Clinicians at Veterans Affairs Hospitals After Prosthetic Joint Infection. *JAMA Netw Open*. Published March 19, 2025. doi:10.1001/jamanetworkopen.2025.1152

Data
Data available: No
